# Temporal and Spatial Requirements of *unplugged*/MuSK Function during Zebrafish Neuromuscular Development

**DOI:** 10.1371/journal.pone.0008843

**Published:** 2010-01-22

**Authors:** Lili Jing, Laura R. Gordon, Elena Shtibin, Michael Granato

**Affiliations:** 1 Department of Cell and Developmental Biology, University of Pennsylvania, Philadelphia, Pennsylvania, United States of America; 2 Department of Biology, University of Pennsylvania, Philadelphia, Pennsylvania, United States of America; Harvard University, United States of America

## Abstract

One of the earliest events in neuromuscular junction (NMJ) development is the accumulation of acetylcholine receptor (AChR) at the center of muscle cells. The *unplugged*/MuSK (muscle specific tyrosine kinase) gene is essential to initiate AChR clustering but also to restrict approaching growth cones to the muscle center, thereby coordinating pre- and postsynaptic development. To determine how *unplugged*/MuSK signaling coordinates these two processes, we examined the temporal and spatial requirements of *unplugged*/MuSK in zebrafish embryos using heat-shock inducible transgenes. Here, we show that despite its expression in muscle cells from the time they differentiate, *unplugged*/MuSK activity is first required just prior to the appearance of AChR clusters to simultaneously induce AChR accumulation and to guide motor axons. Furthermore, we demonstrate that ectopic expression of *unplugged*/MuSK throughout the muscle membrane results in wildtype-like AChR prepattern and neuromuscular synapses in the central region of muscle cells. We propose that AChR prepatterning and axonal guidance are spatio-temporally coordinated through common *unplugged*/MuSK signals, and that additional factor(s) restrict *unplugged*/MuSK signaling to a central muscle zone critical for establishing mid-muscle synaptogenesis.

## Introduction

Synapse formation requires the precise temporal and spatial coordination of pre- and postsynaptic components. Development of the neuromuscular junction (NMJ) is a multi step process that involves a series of reciprocal interactions between the nerve terminal and the muscle [1], [2], [3]. Once muscle cells have differentiated, acetylcholine receptors (AChRs) accumulate at the center of the muscle prior to the arrival of motor growth cones. Motor axons will grow through the region where these prepatterned AChR clusters reside, and induce neuromuscular synapses by incorporating the prepatterned clusters into NMJs [4], [5]. Nerve derived signals disperse prepatterned clusters that are not contacted by the nerve, and thus refine the alignment between the pre- and postsynaptic apparatus [6], [7], [8]. The neuromuscular synapse then forms in the central “end-plate” band of muscle. In mammals, these plaque-shaped clusters are transformed during postnatal development into pretzel-shaped AChR aggregates [1]. In non-mammalian vertebrates, the AChR aggregates do not undergo such elaborate morphological changes.

Genetic studies in mice and fish have established that MuSK is absolutely required for the induction, maturation and maintenance of AChR clusters, as well as for presynaptic development and motor axon guidance [6], [9], [10], [11], [12]. One key question is how MuSK expression and signaling synchronizes pre- and postsynaptic development. In mice, MuSK is expressed in proliferating myoblasts, is up-regulated as myoblasts fuse to form myotubes and persists in adult muscle [13], [14]. Similarly, in zebrafish *unplugged*/MuSK transcripts are first detected in muscle precursors and continue to be expressed into adult stages [11]. Interestingly, *unplugged*/MuSK function can be subdivided into an early, Wnt dependent phase responsible for inducing AChR prepatterning and controlling axonal migration, and a late, Agrin dependent phase that generates neural synapses [15]. While this strongly suggests that *unplugged*/MuSK signaling is required at various developmental time points, it is still unclear if distinct signaling events initiate pre- and postsynaptic development, or if both are initiated simultaneously.

In the mouse embryo, MuSK protein accumulates in the central region of the muscle and co-localizes with AChR clusters throughout NMJ development[14]. While ectopic yet ‘low’ level expression of MuSK throughout the muscle does not alter AChR distribution, ectopic ‘high’ level expression induces ectopic AChR accumulation, suggesting that MuSK expression can ‘induce’ future synaptic sites [16]. In zebrafish, however, ectopic expression of only a combination of *unplugged*/MuSK and *wnt11r* mRNA increased the number and size of prepatterned AChR clusters, suggesting that both ligand and receptor distribution might determine future synaptic sites [15].

Here, we use heat-shock (HS) inducible zebrafish transgenes to examine the spatial and temporal requirements for *unplugged*/MuSK during pre- and postsynaptic development. We find that despite its early expression in muscle precursors, *unplugged*/MuSK activity is first required only when the AChR prepattern starts to form. Moreover, we find that this same time window of *unplugged*/MuSK activity is also critical for directing motor axon pathfinding. In contrast, the requirement for *unplugged*/MuSK signaling to induce neuromuscular synapses later in development is temporally less restricted, which is consistent with the observation that *unplugged*/MuSK can re-establish NMJs after nerve injury. We also find that ubiquitous expression of *unplugged*/MuSK throughout the membrane of the muscle cell properly induces AChR prepattern and neuromuscular synapses in the center of the muscle. Combined, our results support a model by which *unplugged*/MuSK signaling between the 15- and 20-somite stage initiates pre- and postsynaptic development, thereby synchronizing both processes temporally. Our results also suggest that the ability of *unplugged*/MuSK to restrict both AChR clusters and approaching growth cones to the muscle center requires additional factors.

## Results

**Figure 1:**
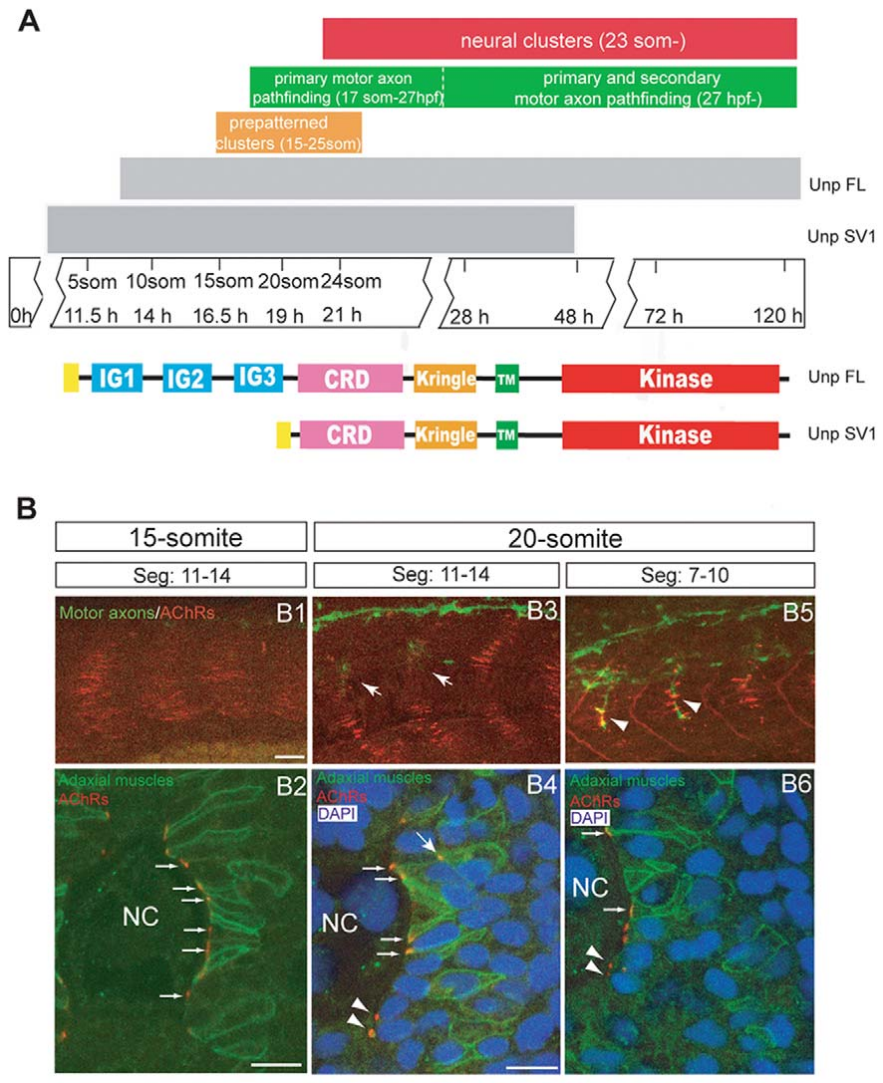
Neuromuscular synaptogenesis in zebrafish. (A) Schematic representation of the different steps during neuromuscular synapse development. *unplugged* SV1 mRNA is expressed transiently from the tailbud stage to 48 hpf, while *unplugged* FL mRNA is expressed from the 10-somite stage and continues to be expressed in adult muscle. Bottom: Domain structure of the *unplugged* FL and SV1 isoforms. (B) Synaptogenesis at the 15- or 20-somite stage. 15-somite (B1) or 20-somite stage (B3 and B4) embryos stained for motor axons (znp-1, green) and AChR clusters (α-BTX, red). In segments 11–14, AChR prepatterned clusters appear at the 15-somite stage before motor neurons have exited the spinal cord (B1); prepatterned clusters coalesce and motor axons (arrows) begin to approach the myotome at the 20-somite stage (B3). In anterior older segments 7–10, motor axons have contacted non-migratory adaxial cells (arrowheads) at the 20-somite stage (B5). (B2, B4, B6) Cross-sectional views of Tg(*smyhc1:mcherry-CAAX*) embryos, which express mCherry specifically on the membrane of adaxial cells, stained for adaxial muscles (anti-DsRed, green), prepatterned clusters (α-BTX, red) and nuclei (DAPI, blue). The same segments as in (B1, B3, B5) were analyzed. (B2) At the 15-somite stage, adaxial muscles remain adjacent to notochord, and prepatterned clusters are localized at the medial side of the cells (small arrows). (B4) At the 20-somite stage, adaxial muscles have migrated laterally. Some prepatterned clusters co-migrate with the adaxial cells (big arrow). Prepatterned clusters appear on the medial side of fast muscle fibers (arrow heads). Prepatterned clusters remain on the medial side of the non-migratory adaxial cells (small arrows). (B6) In older segments 7–10, adaxial muscles have migrated further away; AChR clusters remain on the medial side of non-migratory adaxial cells (small arrows). AChR clusters appear on the medial side of fast muscle fibers (arrow heads). NC: notochord. Scale bars: 20 µM in B; 10 µM in B2 and B4.

We have previously shown that the zebrafish *unplugged* locus generates two MuSK isoforms by alternative splicing, *unplugged* full-length isoform (FL) and *unplugged* splice variant 1 (SV1), which separately control different steps of synaptogenesis. The *unplugged* SV1 isoform controls motor axon guidance and AChR prepatterning, while *unplugged* FL controls the formation of synapses after the nerve contacts the muscle. Consistent with their function, *unplugged* SV1 is transiently expressed during early embryonic development from the tailbud stage up to 48 hpf, while *unplugged* FL expression starts around the 10-somite stage and persists into adult stages (Figure 1A) [11], [15].

To study the functional requirements of *unplugged*/MuSK at high temporal and spatial resolution, we focused on the development of neuromuscular synapses between the 16-somite stage (∼17 hours post fertilization, hpf) when AChR clusters first appear, and 28 hpf, when they form a band of functional neuromuscular synapses (Figure 1B) [4], [5]. The first AChR prepattern forms on adaxial muscle cells, the precursors of the slow twitching muscle fibers [17]. To begin, we examined the precise cellular localization of AChR clusters between the time of AChR prepatterning and synapse formation which coincides with the lateral migration of adaxial cells from their original location next to the notochord to the lateral surface of the myotome. Already before motor axons pioneer into the periphery (15-somite stage), prepatterned AChR clusters are present on the medial side of adaxial muscle cells, initially located next to the notochord (Figure 1B1–B2). At the 20-somite stage, migratory adaxial cells begin their lateral migration, and we noticed that some of these migrating adaxial cells expressed AChR clusters (Figure 1B3–B4). Concomitantly with the onset of adaxial cell migration, fast twitching muscle fibers intercalate into the space between the notochord and adaxial cells [18]. As soon as these intercalating fast muscle fibers contact the notochord, AChR clusters become detectable on their medial side, precisely at the sites where they will make contact with the nerve (Figure 1B3–B6). While fast twitching muscle fibers are present and fully differentiated at around 28pf, we have not been able to detect an obvious AChR prepattern on fast twitching muscle fibers.

### *unplugged*/MuSK Function Is Required Just Prior to the Onset of AChR Prepatterning and Axon Pathfinding

To control when *unplugged*/MuSK is first expressed, we used two previously generated heatshock inducible transgenic lines, Tg(*hsp70l:unplugged SV1myc*) and Tg(*hsp70l:unplugged FLmyc*) crossed into the *unplugged* ^br307/br307^ homozygous background [15]. We had previously shown that HS induction of Tg(*hsp70l:unplugged SV1-myc*) efficiently restores AChR prepatterning and motor axon guidance in *unplugged* embryos. Similarly, Tg(*hsp70l:unplugged FL-myc*) expression in *unplugged* embryos efficiently rescues neural synapses and motor axon pathfinding [15]. In the absence of HS treatment, no AChR prepatterning and neuromuscular synapses are formed and motor axons display pathfinding errors, identical to the phenotype observed in *unplugged* ^br307/br307^ mutants [Figure S1 and15].

**Figure 2:**
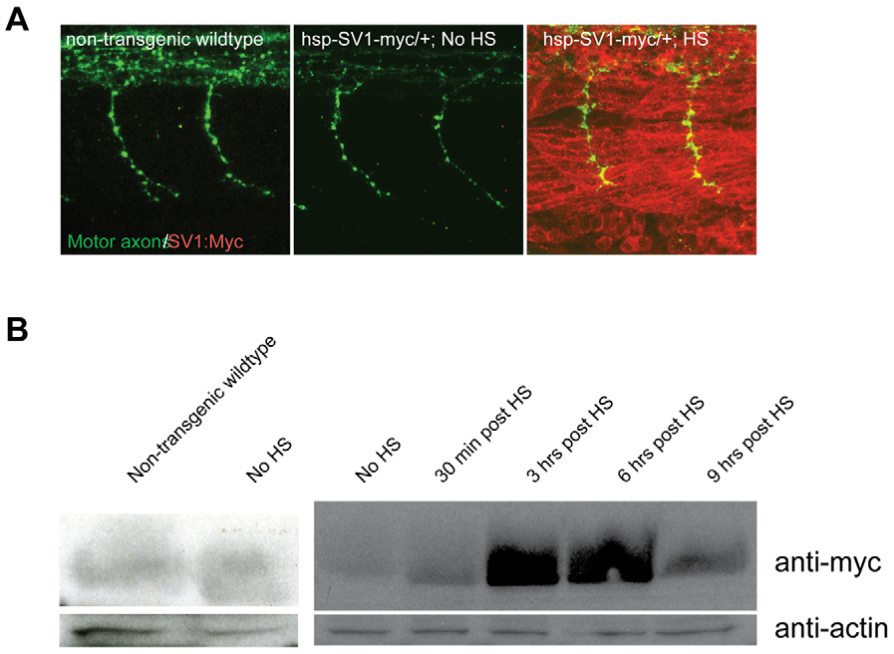
Transgene expression after heat-shock (HS) induction. (A) Lateral views of 26 hpf whole-mount embryos stained with anti znp1 (notor axons, green) and anti myc (red) antibodies. No Myc protein expression is detectable in non-transgenic wildtype and transgenic embryos, but is ubiquitously detectable 10 minutes after a 40-min HS treatment in transgenic embryos. HS treatment was performed at 24hpf for 40 minutes, embryos were fixed and processed at 26 hpf. (B) *unplugged*/MuSK-myc fusion protein was visualized by western blot with an anti-myc antibody (1:500) in non-transgenic wildtype, transgenic non-heat shocked controls, and 30 minutes, 3 hours, 6 hours and 9 hours after a 40-min HS induction of Tg(*hsp70l:unplugged SV1-myc*). An anti-actin antibody (1∶500) is used as a loading control. Tg(*hsp70l:unplugged FL-myc*) shows similar results (data not shown). HS treatment was performed at 24hpf for 40 minutes, embryos were fixed and processed at indicated times post heat shock.

To characterize these transgenic lines further, we determined the time course of transgene induced protein expression. In non-transgenic wildtype or in the absence of HS treatment, Myc protein reflecting transgene expression was not detectable, while 30 minutes of HS treatment resulted in ubiquitous and robust protein expression within 10 minutes following the termination of the heat shock treatment (Figure 2A). Western blot analysis revealed that robust levels of *unplugged*/MuSK-Myc fusion protein are detectable as soon as 30 minutes after HS treatment, and that a single HS treatment resulted in protein expression that lasted for at least 6 hours (Figure 2B). No Myc protein expression was detectable in non-transgenic wildtype controls (Figure 2B). Thus, the two transgenes are effective tools to test when *unplugged*/MuSK function is first required.

**Figure 3:**
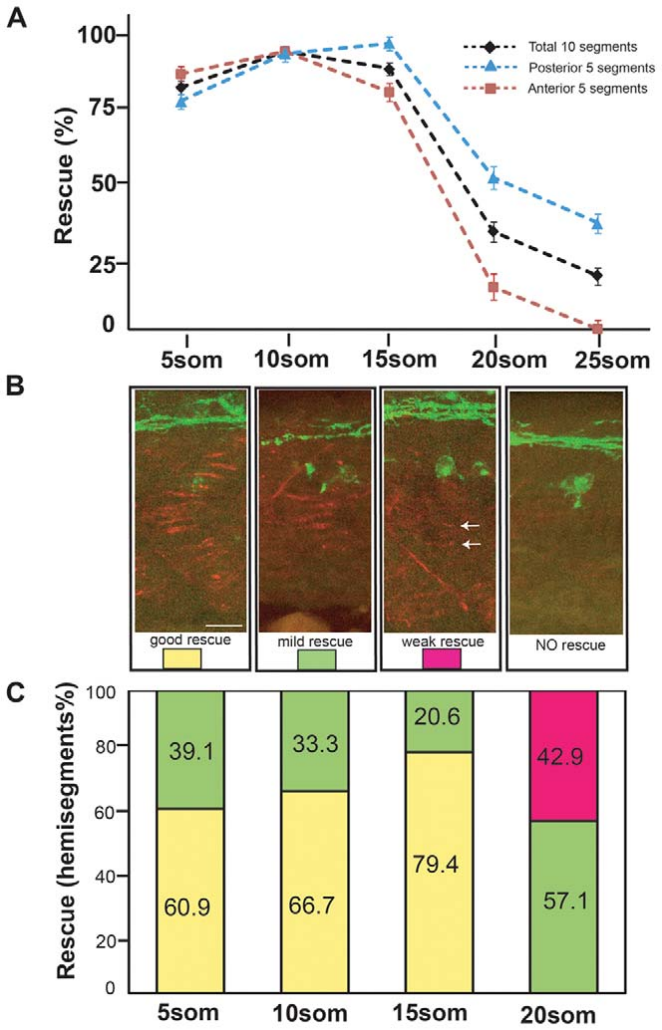
Rescue of motor axon pathfinding and AChR prepatterning by *unplugged*/MuSK at different time points. (A) Temporal series of Tg(*hsp70l:unpFLmyc*) induction to rescue motor axon guidance. Embryos were heat-shocked starting at the indicated time points and analyzed at 27 hpf. The induction of Tg(*hsp70l:unpFLmyc*) expression after the 15-somite stage significantly reduced rescue efficacy (segments 6–15, black; segments 6–10, red; segments 11–15, blue). See Materials and Methods for how rescue efficiency was calculated. 20 hemisegments were analyzed in each embryo, and results from multiple experiments are represented as mean±SEM. (n = 644−1028, average = 744, hemisegments per time point). (B and C) Rescue of AChR prepatterning by Tg(*hsp70l:unplugged SV1-myc*). Embryos were heat-shocked starting at different time points and examined at the 21-somite stage for prepatterned AChR clusters. Only posterior segments (11–15) were analyzed in each embryo (see also Figure S3). (B) Individual hemisegments were scored as ‘good rescue’ (when AChR clusters were present on most muscles), ‘mild rescue’ (when AChR clusters formed on <50% of all muscles, mostly around non-migratory adaxial cells), or ‘weak rescue’ (with smaller prepatterned clusters formed on a few muscles, see arrows). (C) HS treatment of Tg(*hsp70l:unplugged SV1-myc*) embryos at the 20-somite stage significantly reduced AChR rescue efficacy. Results were summarized from multiple experiments (n = 7−20, average = 13, hemisegments per time point). Scale bar: 20 µM.

We then examined when *unplugged*/MuSK is first required for motor axon guidance. For this we analyzed embryos derived from crosses between Tg(*hsp70l:unplugged FL-myc*); *unplugged* ^br307/br307^ and *unplugged* ^br307/br307^ animals, or embryos derived from crosses between Tg(*hsp70l:unplugged SV1-myc*) *unplugged* ^br307/br307^ and *unplugged* ^br307/br307^ animals. 50% of the progeny of this cross are transgenic embryos, and 50% are non-transgenic and were used as an internal control. Transgenic embryos and non-transgenic embryos were HS treated at various starting time points, and somitic segments 6–15 were analyzed at 27 hpf for motor axon pathfinding errors (see Materials and Methods for detail on HS treatments). As shown in Figure 3A, HS induction before or at the 15-somite stage fully restored motor axon guidance. In contrast, HS treatment at or after the 20-somite stage failed to rescue axon guidance defects. Thus, *unplugged*/MuSK is first required between the 15-somite and 20-somite stages to guide motor axons. Since the 10 somitic segments we analyzed represent a relatively broad region, we divided the 10 segments into 5 anterior segments (segments 6–10) and 5 posterior segments (segments 11–15), and analyzed the ability of the transgene to rescue in each of these 5-somite segments. The rescue curve for anterior segments was shifted towards the left and the curve for posterior segments was shifted towards the right (Figure 3A), indicating that the anterior, older segments require *unplugged*/MuSK signaling slightly earlier than the posterior younger segments. This observation is consistent with the fact that embryonic development, including motor axon growth, progresses in a rostral-to-caudal manner. Finally, as we observe identical results using two independent transgenic lines, Tg(*hsp70l:unplugged SV1-myc*, Figure S2) and Tg(*hsp70l:unplugged FL-myc*, Figure 3), respectively, we conclude that *unplugged*/MuSK first becomes essential for axon guidance between the 15-somite and 20-somite stages.

We then determined when *unplugged*/MuSK is first required for AChR prepatterning. For this we examined embryos derived from crosses between Tg(*hsp70l:unplugged SV1-myc*); *unplugged* ^br307/br307^ and *unplugged* ^br307/br307^ animals. We performed HS treatments starting at different times, and examined embryos at the 21-somite stage for the presence/absence of the AChR prepattern (see Materials and Methods for detail about HS induction). At the 21-somite stage, motor axons in the anterior segments (segments 6–10) have already contacted the muscle pioneers and formed synapses, which precludes the analysis of AChR prepatterning. Therefore, we examined the AChR prepattern in the posterior segments only (segments 11–15). To quantify the degree of rescue, each hemisegment was scored as good rescue, mild rescue or weak rescue as shown in Figure 3B and S3A–D (see Figure 3B legend for details on each category).

HS inductions performed before or at the 15-somite stage greatly restored the AChR prepattern, while HS induction at the 20-somite stage rescued AChR prepatterning to a much lesser degree (Figure 3C). To exclude the possibility that the weak rescue at the 20-somite stage was due to the fact that these embryos received fewer HS treatments, we examined the more posterior segments (16–20) in transgenic embryos that were HS induced at the 20-somite stage. The prepatterned clusters in these more posterior segments were partially rescued, and this rescue was much better than that observed in the more anterior segments (11–15) (Figure S3E–H), suggesting that the late onset of *unplugged*/MuSK protein expression rather than the absolute protein level causes the weak rescue in segments 11–15. Thus, *unplugged*/MuSK is first required between the 15-somite and the 20-somite stages to induce the AChR prepattern, which coincides with the time period in which *unplugged*/MuSK is first required to guide motor axons (Figure 3A). This overlapping temporal requirement supports the idea that AChR prepattern and axonal guidance are temporally coordinated through common activation of the *unplugged*/MuSK receptor.

### *unplugged*/MuSK Signaling Remains Competent to Induce Neuromuscular Synapses

**Figure 4:**
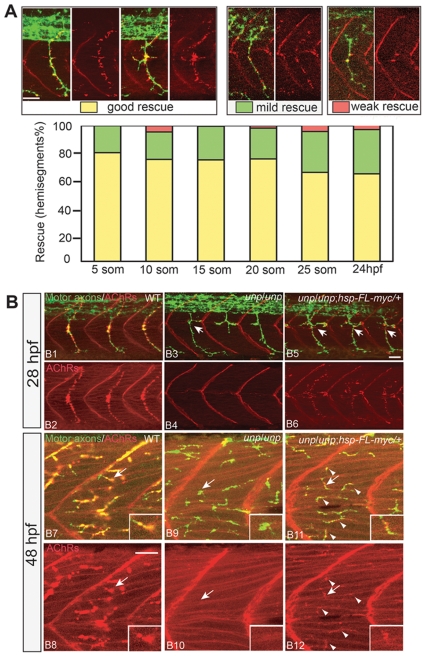
Rescue of neuromuscular synapses by *unplugged*/MuSK. (A) Rescue of neural synapses at different induction times by Tg(*hsp70l:unplugged FL-myc*). Embryos were heat-shocked starting at the indicated time points, fixed at 27hpf, and stained for motor axons (znp-1, green) and AChR clusters (α-BTX, red). Individual hemisegments were scored as ‘good rescue’ (when AChR clusters colocalized with presynaptic nerve ending on most muscle fibers, including along ectopic nerve ending), ‘mild rescue’ (when synapses formed on <50% of all muscles), or ‘weak rescue’ (when synapses formed only on non-migratory adaxial cells). 20 hemisegments were scored in each embryo. Results were summarized from two independent experiments (n = 160−380, average = 280, hemisegments per time point). (B) Tg(*hsp70l:unplugged FL-myc*) rescues neuromuscular synapses at 28 hpf and 48 hpf. Embryos were given a 40-min HS at 28hpf or 48hpf, and examined 30 minutes after the HS treatment. (B1–B6) In transgenic embryos, neuromuscular synapses formed on most muscle fibers and aligned with nerve ending. Arrows point to *unplugged*-characteristic pathfinding errors, indicating that the HS was too late to rescue axon pathfinding. (B7–B12) The single HS treatment at 48 hpf induced synapses in transgenic embryos (arrowheads in B11 and B12), albeit synapses appear smaller than those in wildtype. Inset in each panel is the enlarged image of the neuromuscular synapse pointed by the arrow. Scale bars: 20 µM.

We have previously shown that the AChR prepattern is dispensable for the formation of neuromuscular synapses, thus demonstrating that synaptic development can be divided into distinct functional steps [15]. To further examine the role of *unplugged*/MuSK during the later developmental steps, we analyzed the ability of *unplugged*/MuSK to induce neuromuscular synapses in more detail. For this set of experiments, embryos from Tg(*hsp70l:unplugged FL-myc*); *unplugged* ^br307/br307^ animals crossed to *unplugged* ^br307/br307^ animals were HS treated starting at different times points, and the presence of neuromuscular synapses was scored at 27 hpf (see Materials and Methods for HS treatments). At this time point, AChR clusters are detectable in the center of each muscle fiber where they form focal synapses, as well as aneural AChR at somite boundaries, where once the nerve has extended, myoseptal synapses will form [5], [19]. Individual somitic hemisegments were scored as good, mild or weak rescue depending on the number and size of AChRs clusters that were colocalized with nerve terminals (Figure 4A, see Figure 4 legends for each category of rescue; aneural AChR clusters at the somite boundaries were not included in this analysis). Despite a slight decrease over time, the rescue efficacy of Tg(*hsp70l:unplugged FL-myc*) was similar between the 5-somite stage and 24 hpf. To determine how late during development *unplugged*/MuSK signaling is competent to induce neuromuscular synapses, embryos were HS treated once at 28 hpf or at 48 hpf, and analyzed 30 minutes after HS treatment (Figure 4B). In transgenic embryos which were HS treated at 28 hpf, most if not all neuromuscular synapses were restored, with the majority of postsynaptic AChR clusters correctly aligning with the presynaptic nerve terminals (Figure 4B5–B6). Surprisingly, in transgenic embryos HS treated at 48 hpf, many AChR clusters were present, and properly aligned along most, if not all, nerve endings (arrowheads in Figure 4B11–B12). However, we noticed that these neural AChR clusters appeared smaller and less numerous than those in wildtype embryos. To determine if the small size of rescued synapses at 48 hpf is due the reduced competence of muscle fibers to form AChRs or due to a low level of protein resulting from only one HS treatment, we repeated this experiment but analyzed the embryos 3 hrs after HS treatment. As shown in Figure S3I-L, postsynaptic AChR clusters of wildtype size were aligned with the presynaptic nerve terminals, consistent with the well-documented ability of regenerating axons to restore NMJs after nerve injury in adult muscle [20]. Thus, like in mammals, *unplugged*/MuSK signaling can induce neural AChR clustering late in development, presumably with the condition that nerve-derived Agrin is present.

### Ectopic *unplugged*/MuSK Expression Results in Localized AChR Accumulation

In the mouse, MuSK protein colocalizes with AChR clusters to a narrow zone on the muscle surface [14]. Increasing MuSK protein expression via transgenesis broadens the domain of AChR accumulation and nerve growth, which has led to a model by which MuSK determines where future synapses form [16]. This model also predicts that *in vivo*, MuSK self-actives preferentially in the muscle center, thus requiring no endogenous ligand [21]. Yet, the low-density lipoprotein receptor-related protein 4 (LRP4) has recently been shown to function as a co-receptor with MuSK to bind Agrin and induce neural synapses [22], [23], [24]. LRP4, whose extracellular domain is similar to the Wnt coreceptor LRP5/6 protein, is also required for AChR prepattern [22]. Moreover, in zebrafish the secreted Wnt11r protein functions as an *unplugged*/MuSK ligand and is required to initiate AChR prepatterning [15]. Combined, these results raise the question as to whether MuSK localization is instructive or permissive.

**Figure 5:**
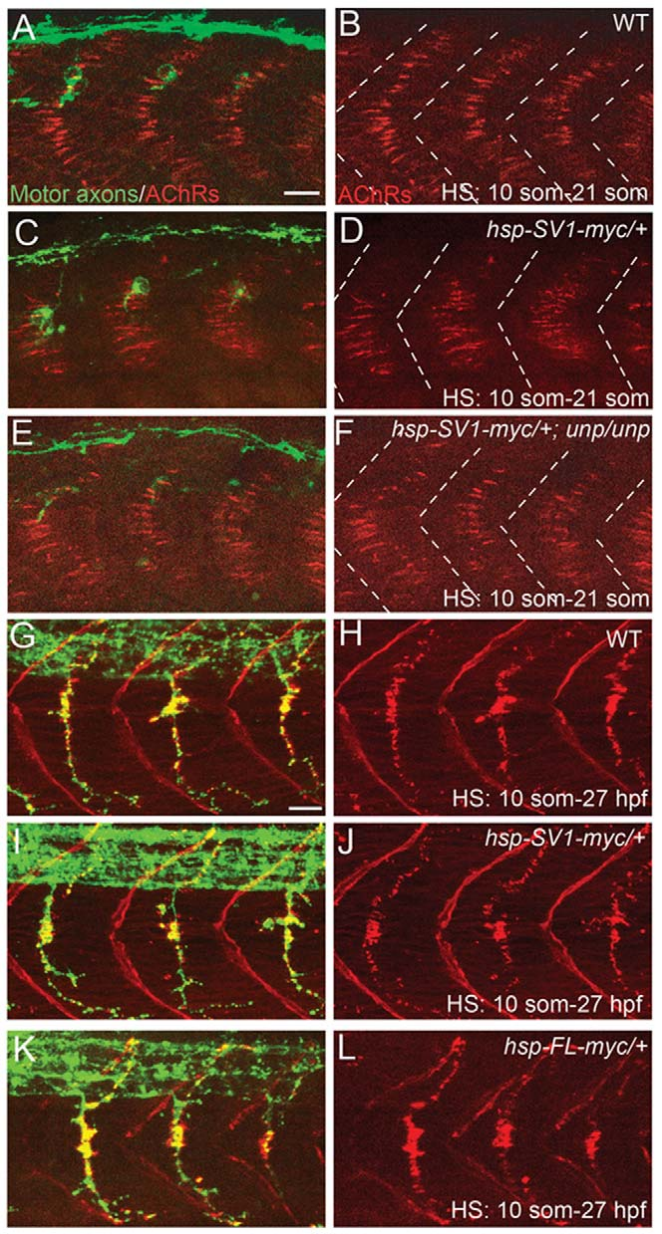
Overexpression of *unplugged*/MuSK in WT embryos does not perturb synaptogenesis. 21-somite stage (A–F) or 27 hpf (G–L) embryos after HS treatment. (A–F) Wildtype (WT) embryos, Tg(*hsp70l:unplugged SV-1myc*) embryos, and Tg(*hsp70:unplugged SV1-myc*); *unplugged^br307/br307^* embryos received HS from the 10- to 21-somite stage The AChR prepatterning zone appears slightly expanded in (D) compared to (B and F). White dashed lines mark the boundaries of each segment. (G–L) WT embryos, *hsp70l:unplugged SV-myc* embryos, (I and J) or *hsp70l:unplugged FL-myc* embryos (K and L) were heat shocked from the 10-somite stage until 27 hpf. Motor axons and neuromuscular synapses appear normal in the embryos expressing the transgene (I–L). Scale bars: 20 µM.

To examine this question, we used the transgenic lines and HS treatments that were sufficient to restore axonal guidance and synaptogenesis in *unplugged* mutants. At present, antibodies that recognize the zebrafish *unplugged*/MuSK protein are not available, precluding us from determining its endogenous levels and cellular localization in muscle. However, based on rescue experiments and on Western Blot analysis (Figure 2B), *unplugged*/MuSK protein levels in HS treated transgenic embryos are higher than those of muscle actin, suggesting that in these transgenic embryos muscle cells express exceedingly high levels of functional *unplugged*/MuSK protein. Analysis of HS treated embryos revealed that transgenic overexpression of Tg(*hsp70l:unplugged SV1-myc*) in wildtype or *unplugged* mutant embryos did not affect the location of the AChR prepattern (Figure 5A–F), or motor axons guidance (Figure 5I and J). Although the AChR prepatterning appeared slightly expanded, it was still restricted to the center of the somitic segments (Figure 5C and D). Similarly, overexpression of Tg(*hsp70l:unplugged FL-myc*) did not affect the formation of neuromuscular synapses and motor axon outgrowth (Figure 5K and L).

**Figure 6:**
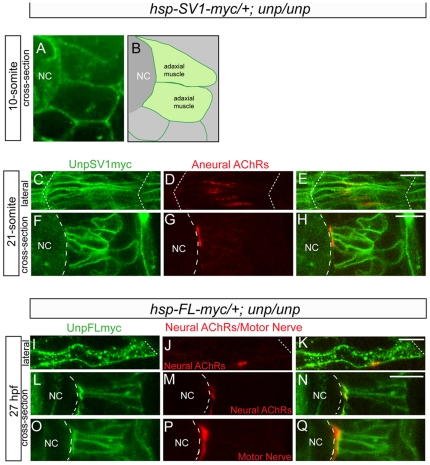
Cellular localization of exogenous *unplugged*/MuSK protein and AChR clusters. (A) Tg(*hsp70l:unplugged SV1-myc*); *unplugged* ^br307/br307^ embryos at the 10-somite stage after the HS treatment, stained for anti-Myc Ab (green). Prior to the appearance of AChR clusters Unplugged SV1 protein is distributed evenly on the surface of adaxial muscle cells. (B) Diagram of A. (C–H) Tg(*hsp70l:unplugged SV1-myc*); *unplugged* ^br307/br307^ embryos at the 21-somite stage after the HS treatment, stained for anti-Myc Ab (green) and AChR clusters (α-BTX, red). Lateral views (C–E) and cross-sectional views (F–H) show that Unplugged SV1 protein is distributed throughout the surface of muscle cells, but prepatterned clusters are localized at the center and the medial side of the cell surface. (I–Q) Tg(*hsp70l:unplugged FL-myc*); *unplugged* ^br307/br307^ embryos at 27 hpf after HS treatment, stained for anti-Myc Ab (green, I–Q) and AChR clusters (α-BTX, red, J,K,M,N) or znp1(red, P–Q). Unplugged FL protein is expressed throughout the cell membrane as shown in lateral views (I–K), or cross-sectional views, (L–Q). Neural AChR clusters and motor axons are located at the center and the medial side of the cell. Non-migratory adaxial cells at the horizontal myoseptum were imaged. White dashed lines mark the boundaries of the somite in (C–E, I–K) or indicate the position of notochord in (A, B, F–H, L–Q). NC, notochord. Scale bars: 10 µM.

To examine the precise localization of AChR clusters in relation to the expression of exogenous *unplugged* SV1 and FL proteins in more detail, we visualized exogenous protein distribution using an anti-Myc antibody. To exclude the interference from the endogenous *unplugged*/MuSK protein, we examined the exogenous proteins in *unplugged* null mutants. Prior to the appearance of the AChR prepatterning (10 somite stage), *unplugged* -Myc proteins were ubiquitously distributed on the surface of presumptive adaxial muscle cells (Figure 6A, B). During the period of AChR prepatterning (21 somite stage), *unplugged* SV1-Myc protein was also ubiquitously distributed on the membrane of muscle cells (Figure 6C–H). Despite its ubiquitous membrane localization, prepatterned AChR clusters were restricted to medial membranes and to the muscle center, identical to the wildtype situation (Figure 6C–H). Similarly, *unplugged* FL-Myc proteins were ubiquitously localized on the membrane of muscle cells, while neuromuscular synapses were again restricted to medial membranes and to the center of muscle cells (Figure 6I–Q). Thus, in zebrafish muscle exceedingly high levels of *unplugged*/MuSK protein ubiquitously distributed on muscle cell membranes still direct AChR prepatterning, synaptogenesis and motor axon growth to the muscle center.

## Discussion

The formation of neuromuscular synapses requires coordinated pre- and postsynaptic development. The *unplugged*/MuSK receptor tyrosine kinase is an essential gene expressed in all skeletal muscle to restrict presynaptic nerve growth and postsynaptic AChR accumulation to the muscle center. Despite its prominent role in this process, it has remained unclear how *unplugged*/MuSK signaling initiates this highly coordinated alignment of pre- and postsynaptic structures, both temporally and spatially. For example, it is unclear if pre- and postsynaptic development is initiated by sequential or by common *unplugged*/MuSK signaling events. Similarly, the presence of endogenous ligands that initiate *unplugged*/MuSK signaling has raised the question as to whether *unplugged*/MuSK receptor localization is sufficient or permissive to mark the sites of future synapses *in vivo*. Here, we show that *unplugged*/MuSK is first required to induce AChR prepatterning and to mediate axon pathfinding during a common, narrow time window, which provides further evidence that both processes are coordinated through a common *unplugged*/MuSK signaling event. Using the same transgenic approach, we expand the *unplugged*/MuSK expression domain to the entire muscle surface, but find that AChR prepattern and synapses remain restricted to the central muscle region, consistent with the idea that *in vivo* additional, extrinsic and/or muscle intrinsic mechanisms restrict *unplugged*/MuSK signaling to the mid-muscle region.

### A Common *unplugged*/MuSK Signaling Event to Coordinate Pre- and Postsynaptic Development

In zebrafish, the first prepatterned AChR clusters form on adaxial muscles which are slow twitching muscle progenitors (Figure 1). The mRNA expression of *unplugged*/MuSK in adaxial cells is first detected at the tail bud stage (∼10 hpf), just before the onset of somitogenensis [11]. We find that *unplugged*/MuSK activity is dispensable from this time point until the 15-somite stage (∼16 hpf), and becomes essential by the 20-somite stages (∼19 hfp, in segments 11–15) to initiate AChR prepatterning. This is about 6 hours after the onset of *unplugged*/MuSK expression in adaxial cells, and just when the AChR prepattern begins to form. Although it is unclear to which extent *unplugged*/MuSK mRNA expression reflects protein expression, the first functional requirement of *unplugged*/MuSK 6 hours after the onset of expression favors the idea that initiation of *unplugged*/MuSK signaling requires a ligand, *wnt11r*, even at these early stages. Future studies using antibodies against *unplugged*/MuSK and *wnt11r* will be essential to address how both proteins are initially localized.

Intriguingly, *unplugged*/MuSK is required during the same time window to restrict motor axons to the muscle center, about 2–3 hours before the motor axons actually contact the muscle (Figure 1). These results are consistent with the idea that a common *unplugged*/MuSK signaling event temporally initiates and thereby coordinates pre- with postsynaptic development. Although we have not examined *unplugged*/MuSK signaling directly, i.e. by assessing receptor phosphorylation, the data is consistent with our model that *unplugged*/MuSK signaling through *dishevelled* primarily functions to organizes a central muscle zone to which AChR prepatterning and growth cone guidance is restricted [15].

At the same time, the data also raises the question as to why there is a lag time between the first time *unplugged*/MuSK is required and the onset of axonal growth. We had previously shown that signaling downstream of *unplugged*/MuSK and *dishevelled* diverges, as the intracellular linker protein Rapsyn, essential for AChR accumulation, is dispensable for axon guidance [11], [15]. Furthermore, *unplugged*/MuSK expressing adaxial muscle cells produce extracellular matrix (ECM) components, which accumulate in the center of muscle fibers, flanking the approaching motor axon [11], [25]. Two of these ECM components, Tenascin C and chondroitin sulfate proteoglycans (CSPG) are known regulators of axonal outgrowth, and in *unplugged* mutants, they fail to accumulate at the muscle center [11], [25]. Thus, it is conceivable that *unplugged*/MuSK activation leads to a rapid accumulation of AChRs via the intracellular linker Rapsyn, while the accumulation of extracellular components at the muscle center is independent of Rapsyn and occurs at a slower rate, possibly due to the time required for secretion and/or additional modifications of these ECM components.

### In Zebrafish *unplugged*/MuSK Expression Is Not Sufficient to Predict Synaptic Sites

In mice, MuSK expression is first detectable in proliferating myoblasts [13]. From a series of experiments it has been proposed that the early expression of MuSK protein in developing myotube determines the future synaptic sites in the central band of the muscle [21]. Muscle grows by the fusion of myoblasts symmetrically on either side of developing myotube. MuSK is first expressed in the central nuclei of the myotube, where it becomes activated stochastically. Activated MuSK recruits more MuSK protein and stimulates MuSK expression through positive feedback loops, which further enhances MuSK signaling at the central region of the muscle [16], [21]. Consistent with this model, ectopic expression of MuSK at high levels induces ectopic AChR accumulation [16].

Recently, LRP4 has been shown to function as a MuSK co-receptor for Agrin to induce neural synapses [22], [23], [24]. Moreover, in zebrafish, the secreted Wnt11r protein functions as a *unplugged*/MuSK ligand required to initiate AChR prepatterning [15]. Combined, these results are more consistent with the idea that endogenous ligands initiate MuSK activation in mouse and fish embryos. Finally, low level MuSK protein expression throughout the diaphragm muscle restored AChR prepattern in the center of muscle fibers [16], supporting the notion that additional factors play a role in determining the synaptic sites. The results presented here demonstrate that exceedingly high expression of functional *unplugged*/MuSK-Myc protein ubiquitously on the muscle membrane induced prepatterned AChR clusters in the muscle center, and restricted axons to the muscle center (Figure 6C–H). Although we show that the levels of *unplugged*/MuSK protein are comparable to those of muscle actin, and hence significantly above the expected endogenous *unplugged*/MuSK levels, we can not exclude the possibility that even higher levels of exogenous *unplugged*/MuSK protein are required to induce ectopic AChR clusters and perturb axonal growth. Alternatively, anatomical and developmental differences might account for the differences observed between overexpression of *unplugged*/MuSK in mice and fish embryos. For example, unlike polynucleated mouse diaphragm muscle which grow at their ends (see above), zebrafish adaxial muscle cells are mononucleated, and they achieve their length by elongating in an anterior-posterior dimension until they span the entire length of the somite [17].

Nonetheless, our results suggest that *unplugged*/MuSK alone is not sufficient to induce AChR prepatterning and synapse formation, and is consistent with our previous results demonstrating that *unplugged*/MuSK and *wnt11r* are mutually required [15]. It is likely that zebrafish *unplugged* protein is localized to the muscle center, as has been demonstrated in the mouse [14], and that in addition secreted Wnt11r protein is presented exclusively to this domain. This could be achieved via localized, muscle intrinsic co-receptor proteins, for example by Lrp4 or other Lrp family members [22], [23], [24]. Obviously, this does not exclude the presence of additional, localized muscle intrinsic factors such as Dok-7 [26], [27], and further studies are required to distinguish between these possibilities.

### The Role of *unplugged*/MuSK Signaling in Coordinating Developmental Processes

We have previously shown that the *unplugged*/MuSK locus generates two distinct *unplugged*/MuSK isoforms that require two different ligands to separately control the early and late stages of synaptogenesis [11], [15]. The data presented here suggest that the early Wnt dependent function of *unplugged*/MuSK to establish a central muscle zone for AChR prepatterning and motor axons is temporally restricted, while its later, Agrin dependent function to induce neuromuscular synapses, is temporally less restricted. This is reminiscent of the temporal requirement of the *C. elegans* SAD-1 kinase in neuronal polarization and synapse organization [28]. Similar to *unplugged/*MuSK function in synaptogenesis, SAD-1 activity is strictly required during the time period when neurons undergo polarization. The temporal requirement for SAD-1 is less stringent in subsequent synaptic organization. Moreover, SAD-1 can re-establish synaptic organization during the maintenance step. Interestingly, like *unplugged*/MuSK, SAD-1 regulates neuronal polarity and synaptic organization through different genetic pathways [29]. These general similarities might suggest a common theme in that developmental processes that follow each other employ a common signaling protein that is able to interface with different pathways to ensure coordinated progression between developmental process.

## Materials and Methods

### Ethics Statement

All experiments were conducted according to an Animal Protocol fully approved by the University of Pennsylvania Institutional Animal Care and Use Committee (IACUC) on February 15, 2008, protocol number 459800.Veterinary care is under the supervision of the University Laboratory Animal Resources (ULAR) of the University of Pennsylvania.

### Zebrafish Genetics

All the fish were in the Tü or TLF genetic background. *unplugged* ^tbr307^ null alleles were used for all experiments.

### Transgenes

Tg(*smyhc1*:mCherry-CAAX) was generated by microinjection of DNA as previously described in [30]. Generation of Tg(*hsp70l:unplugged SV1-myc*)p1, Tg(*hsp70l:unplugged FL-myc*)p1 was done following a standard protocol as previously reported in [15].

### Heat-Shock Treatments

The embryos from the cross of Tg(*hsp70l*:*SV1(or FL)-myc*); *unplugged* ^br307/br307^ to *unplugged* ^br307/br307^ were kept at 28°C to the desired stage before HS. Embryos were then placed in 100 µl E3 medium in a single well of 96-well PCR plate to receive HS. HS treatments were performed as follows unless otherwise noted.

#### HS treatments for motor axon pathfinding

To minimize non-specific HS effects, embryos were exposed to two HS treatments (40 minutes at 38°C, followed by a 2.5-hour period at 28°C, and another 40 minute HS treatment at 38°C). This treatment had little to no effect on non-transgenic embryos, but efficiently rescued motor axon pathfinding in transgenic embryos (Figure S1). Tg(*hsp70l:unplugged FL-myc*) restores motor axon pathfinding more efficiently than Tg(*hsp70l:unplugged SV1-myc*) (Figure S1). Thus, Tg(*hsp70l:unplugged FL-myc*) was used to analyze the temporal requirement for *unplugged*/MuSK during motor axon guidance. Embryos were heat shocked starting at different time points. Following HS, embryos were transferred from the PCR plate to a Petri dish and kept at 28°C until 27 hpf.

#### HS treatments for AChR prepattern and neuromuscular synapses

To obtain maximum rescue of the AChR prepattern and neuromuscular synapses, repeated HS treatments were performed. Embryos were repeatedly heat shocked at 38°C for 40 minutes with 2.5-hour intervals starting at different times until they reached the 21-somite stage (for prepatterning) or 27 hpf (for neuromuscular synapses). Tg(*hsp70l:unplugged SV1-myc*) was used to examine the temporal requirement of *unplugged*/MuSK for AChR prepattern. Tg(*hsp70l:unplugged FL-myc*) was used to analyze the temporal requirement for neuromuscular synapses. Transgenic embryos were identified by genotyping using the following primers: 5′TGACCAGATGCTCAAATCTGGTCTTTC3′ (forward) and 5′ATTAAGCTAGCGGTGAGGTCGCCCTA3′(reverse).

### Analysis of Heat Shock Treated Embryos to Determine Rescue Efficiency

Maximum rescue under various conditions resulted in 81.2% of all hemisegments displaying wildtype axons, which were set to 100% rescue. The ‘weakest’ rescue resulted in 22.6% of all hemisegments displaying wildtype axons, which was set to 0% rescue. The percentile of rescue for each time point was determined by using the following formula: (Y-22.6)X100/58.6, where Y = the percentile of wildtype axons in the transgenic embryos heat-shocked at that specific starting time point, and 58.6 = (81.2−22.6). 20 hemisegments were analyzed in each embryo. Results were obtained from multiple experiments and represented as mean±SEM. (n = 644−1028, average = 744, hemisegments per time point).

### Whole-Mount Inmmunocytochemistry

Embryos were fixed and stained as previously described [31]. To label AChRs, embryos were treated with 0.1% collagenase for 5 minutes (20-somite), 7 minutes (27 hpf) or 45 minutes (48 hpf), as previously described [19]. Antibodies and dilutions were used as follows: znp-1 (1∶200, DSHB), myc (9E10, 1∶1000, Covance), Alexa488 conjugated α-bungarotoxin (1∶100, Molecular Probes, Eugene, OR). Embryos were mounted in Vectashield (Molecular Probe) and imaged with an LSM510 (Zeiss) confocal microscope.

### Western Blotting

24 hpf embryos from the cross between Tg(*hsp70l*:*unplugged SV1(FL)-myc*); *unplugged* ^br307/br307^ and *unplugged* ^br307/br307^ adults were heat shocked at 38°C for 40 minutes. After HS treatment, embryos were transferred into lysis buffer (10 mM Tris pH 7.5, 150 mM NaCl, 5 mM EDTA, 1% NP-40, 10% glycerol) and then quickly frozen in liquid nitrogen. The head of the embryo was used for genotyping. For Western Blot analysis, lysates from five embryos were pooled, and the protein concentration was determined using a standard Bradford assay (Biorad). The same amount of total proteins from each sample was loaded on SDS-gel (10%) and blotted with anti-myc antibody (9E10, 1∶1000, Covance) and anti-actin antibody (1∶3000,I tried 1∶1000, 1∶200, got slightly stronger band, but higher background). The secondary antibodies were HRP-conjugated goat anti-mouse and goat anti-rabbit (1∶5000, GE healthcare). The blots were detected by ECL Plus chemiluminescent detection system (GE healthcare).

## Supporting Information

Figure S1 (A) Lateral views of 27-hpf wildtype, *unplugged* and Tg(*hsp70l:unplugged SV1-myc*); *unplugged^br307/br307^* embryos stained for motor neurons (znp-1). Motor axons in wildtype embryos extend into the ventral myotome after the choice point (arrowheads). *unplugged* motor axons form lateral branches (arrows), or stall (data now shown) at the choice point. Axonal pathfinding defects were rescued in Tg(*hsp70l:unplugged SV1-myc*); *unplugged^br307/br307^* embryos after the appropriate HS treatment. (B) Quantification of motor axonal phenotypes. Embryos were heat-shocked from the 10-somite stage to 27 hpf. HS treatment did not have obvious effect on motor axon pathfinding in wildtype and *unplugged* embryos (columns 1–3). In the absence of HS treatment, motor axons remain disrupted in Tg(*hsp70l:unplugged FL-myc*); *unplugged^br307/br307^* (column 4) and Tg(*hsp70l:unplugged SV1-myc*); *unplugged^br307/br307^* (data not shown) embryos. After HS treatment, motor axons were significantly rescued in transgenic embryos (columns 5–6). 20 hemisegments in each embryo were scored. Results are expressed as the average of multiple embryos (n≥20).
(0.54 MB TIF)

Figure S2 Tg(*hsp70l:unplugged SV1-myc*); *unplugged^br307/br307^* embryos were heat-shocked starting at the indicated times points, and examined at 27 hpf for motor axon pathfinding. Results were summarized from one experiment, analyzed as in Figure 3A, and represented as mean±SEM. 20 hemisegments were scored in each embryo (n = 180−400, average = 328, hemisegments per time point).
(0.14 MB TIF)

Figure S3 (A-D) Representative images for the AChR prepattern rescue in 21-somite Tg(*hsp70l:unplugged SV1-myc*); *unplugged^br307/br307^* embryos after HS treatments. Embryos were stained for motor neurons (znp-1, green) and AChRs (α-BTX, red). Segments 11-15 were imaged in each embryo. (E-H) Tg(*hsp70l:unplugged SV1-myc*); *unplugged^br307/br307^* embryos were heat-shocked for 40 minutes at the 20-somite stage and examined at the 21-somite stage for motor neurons and AChRs. AChR prepatterning was not well rescued in segments 11-15 (E and G). Posterior segments 16-20 from the same embryo display better rescue of the prepatterning (F and H). (G and H) Enlarged images of the highlighted segments in E and F. Arrow in (H) indicate the large wildtype-like prepatterned clusters. Arrowheads in (G) mark the small punctate clusters. Scale bars: 20 µM. (I-L) Rescue of neural synapses at 48hpf by Tg(*hsp70l:unplugged FL-myc*). Embryos were heat-shocked for 40 minutes at 48hpf, fixed at 51hpf and stained for motor axons (znp-1, green) and AChR clusters (α-BTX, red). (I and J) Neural synapses induced by heat shock treatment of Tg(*hsp70l:unplugged FL-myc*); *unplugged^br307/br307^* embryos persisted for 3 hours following the heat shock. (K and L) No neural synapses were induced in *unplugged^br307/br307^* embryos lacking the transgene. Arrows in I and K point to rescued and non-rescued synapses respectively with insets showing enlarged views.
(1.07 MB TIF)
