# Supplementary figures and images for: Temporal and Spatial Requirements of unplugged/MuSK Function during Zebrafish Neuromuscular Development
Source: PLoS One. 2010 Jan 22;5(1):e8843. doi: 10.1371/journal.pone.0008843 (PMC2809748; doi:10.1371/journal.pone.0008843)

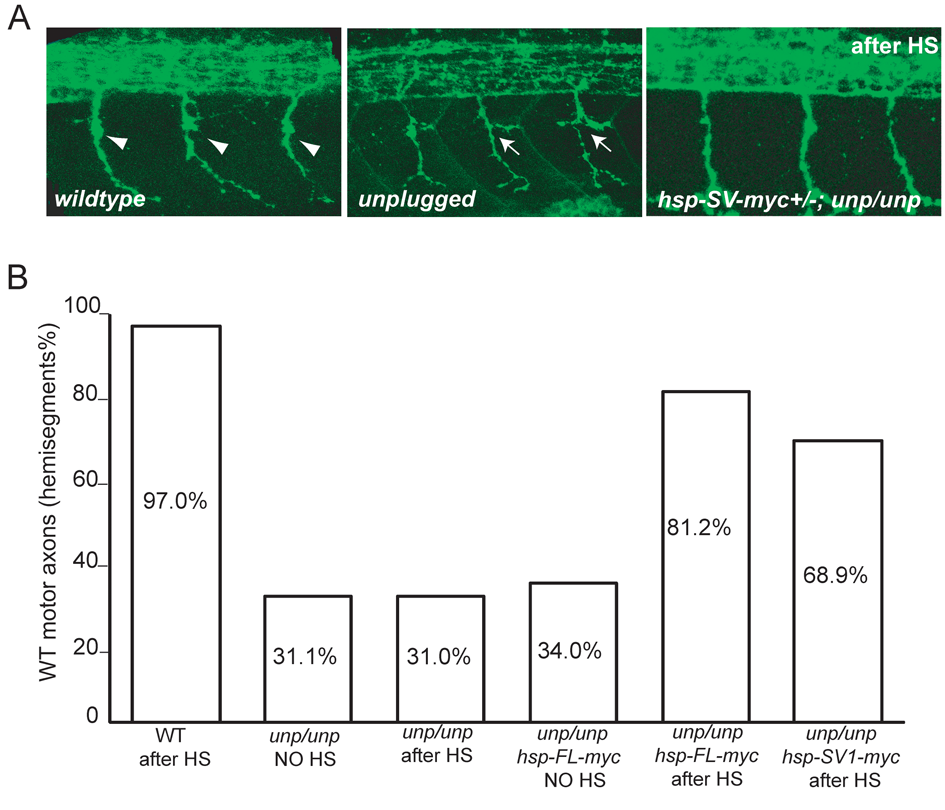

Supplement: Figure S1 — (A) Lateral views of 27-hpf wildtype, unplugged and Tg(hsp70l:unplugged SV1-myc); unpluggedbr307/br307 embryos stained for motor neurons (znp-1). Motor axons in wildtype embryos extend into the ventral myotome after the choice point (arrowheads). unplugged motor axons form lateral branches (arrows), or stall (data now shown) at the choice point. Axonal pathfinding defects were rescued in Tg(hsp70l:unplugged SV1-myc); unpluggedbr307/br307 embryos after the appropriate HS treatment. (B) Quantification of motor axonal phenotypes. Embryos were heat-shocked from the 10-somite stage to 27 hpf. HS treatment did not have obvious effect on motor axon pathfinding in wildtype and unplugged embryos (columns 1–3). In the absence of HS treatment, motor axons remain disrupted in Tg(hsp70l:unplugged FL-myc); unpluggedbr307/br307 (column 4) and Tg(hsp70l:unplugged SV1-myc); unpluggedbr307/br307 (data not shown) embryos. After HS treatment, motor axons were significantly rescued in transgenic embryos (columns 5–6). 20 hemisegments in each embryo were scored. Results are expressed as the average of multiple embryos (n≥20). (0.54 MB TIF) [file pone.0008843.s001.tif]

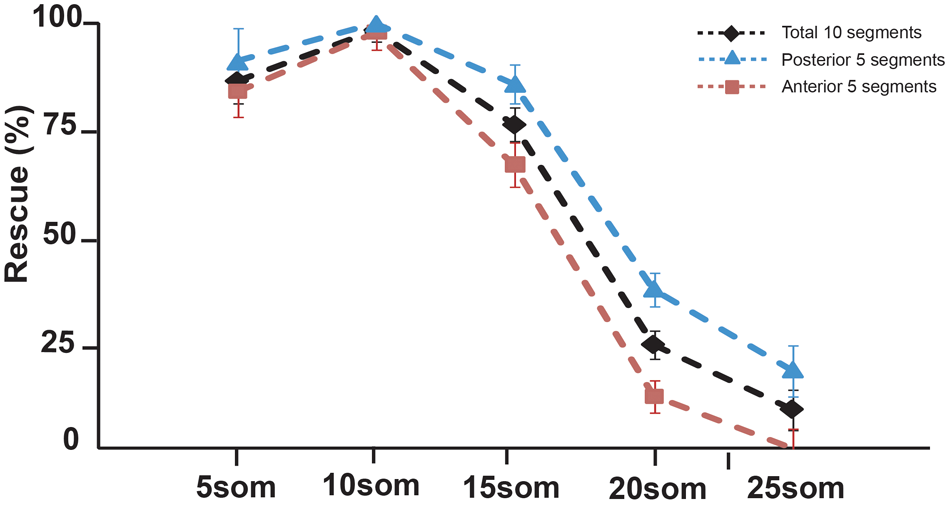

Supplement: Figure S2 — Tg(hsp70l:unplugged SV1-myc); unpluggedbr307/br307 embryos were heat-shocked starting at the indicated times points, and examined at 27 hpf for motor axon pathfinding. Results were summarized from one experiment, analyzed as in Figure 3A, and represented as mean±SEM. 20 hemisegments were scored in each embryo (n = 180−400, average = 328, hemisegments per time point). (0.14 MB TIF) [file pone.0008843.s002.tif]

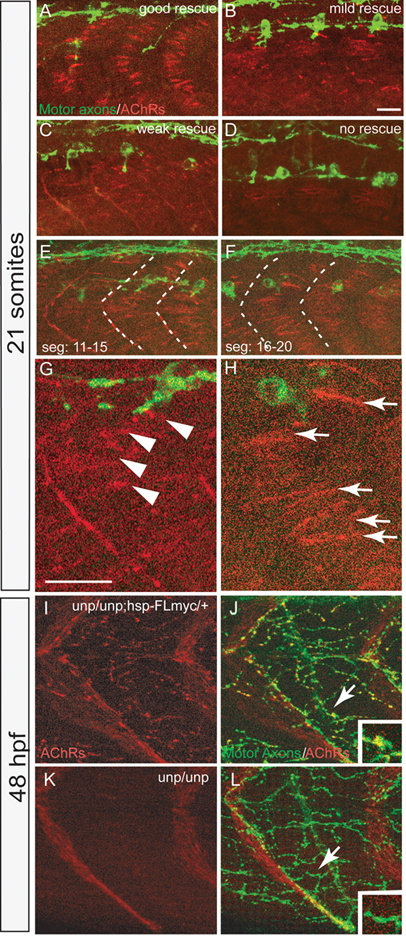

Supplement: Figure S3 — (A-D) Representative images for the AChR prepattern rescue in 21-somite Tg(hsp70l:unplugged SV1-myc); unpluggedbr307/br307 embryos after HS treatments. Embryos were stained for motor neurons (znp-1, green) and AChRs (α-BTX, red). Segments 11-15 were imaged in each embryo. (E-H) Tg(hsp70l:unplugged SV1-myc); unpluggedbr307/br307 embryos were heat-shocked for 40 minutes at the 20-somite stage and examined at the 21-somite stage for motor neurons and AChRs. AChR prepatterning was not well rescued in segments 11-15 (E and G). Posterior segments 16-20 from the same embryo display better rescue of the prepatterning (F and H). (G and H) Enlarged images of the highlighted segments in E and F. Arrow in (H) indicate the large wildtype-like prepatterned clusters. Arrowheads in (G) mark the small punctate clusters. Scale bars: 20 µM. (I-L) Rescue of neural synapses at 48hpf by Tg(hsp70l:unplugged FL-myc). Embryos were heat-shocked for 40 minutes at 48hpf, fixed at 51hpf and stained for motor axons (znp-1, green) and AChR clusters (α-BTX, red). (I and J) Neural synapses induced by heat shock treatment of Tg(hsp70l:unplugged FL-myc); unpluggedbr307/br307 embryos persisted for 3 hours following the heat shock. (K and L) No neural synapses were induced in unpluggedbr307/br307 embryos lacking the transgene. Arrows in I and K point to rescued and non-rescued synapses respectively with insets showing enlarged views. (1.07 MB TIF) [file pone.0008843.s003.tif]
